# Interstitial lung diseases in children

**DOI:** 10.1186/1750-1172-5-22

**Published:** 2010-08-20

**Authors:** Annick Clement, Nadia Nathan, Ralph Epaud, Brigitte Fauroux, Harriet Corvol

**Affiliations:** 1 Pediatric Pulmonary Department, Reference Center for Rare Lung Diseases, AP-HP, Hôpital Trousseau, Inserm UMR S-938; Université Pierre et Marie Curie-Paris 6, Paris, F-75012 France

## Abstract

Interstitial lung disease (ILD) in infants and children comprises a large spectrum of rare respiratory disorders that are mostly chronic and associated with high morbidity and mortality. These disorders are characterized by inflammatory and fibrotic changes that affect alveolar walls. Typical features of ILD include dyspnea, diffuse infiltrates on chest radiographs, and abnormal pulmonary function tests with restrictive ventilatory defect and/or impaired gas exchange. Many pathological situations can impair gas exchange and, therefore, may contribute to progressive lung damage and ILD. Consequently, diagnosis approach needs to be structured with a clinical evaluation requiring a careful history paying attention to exposures and systemic diseases. Several classifications for ILD have been proposed but none is entirely satisfactory especially in children. The present article reviews current concepts of pathophysiological mechanisms, etiology and diagnostic approaches, as well as therapeutic strategies. The following diagnostic grouping is used to discuss the various causes of pediatric ILD: 1) exposure-related ILD; 2) systemic disease-associated ILD; 3) alveolar structure disorder-associated ILD; and 4) ILD specific to infancy. Therapeutic options include mainly anti-inflammatory, immunosuppressive, and/or anti-fibrotic drugs. The outcome is highly variable with a mortality rate around 15%. An overall favorable response to corticosteroid therapy is observed in around 50% of cases, often associated with sequelae such as limited exercise tolerance or the need for long-term oxygen therapy.

## Definition

Interstitial lung disease (ILD) in infants and children represents a heterogeneous group of respiratory disorders that are mostly chronic and associated with high morbidity and mortality (around 15%) [1,2]. These disorders are characterized by inflammatory and fibrotic changes that affect alveolar walls. Typical features of ILD include the presence of diffuse infiltrates on chest radiograph, and abnormal pulmonary function tests with evidence of a restrictive ventilatory defect (in older children) and/or impaired gas exchange [3].

## Classification

There have been many different approaches to the classification of ILD, with major shifts based on clinical investigation, improvement in chest imaging, and collaboration with pathologists. In 1998, Katzenstein and Myers proposed four histopathologically distinct subgroups of idiopathic interstitial pneumonias: usual interstitial pneumonia (UIP), desquamative interstitial pneumonia (DIP) and a closely related pattern termed respiratory bronchiolitis-associated ILD, acute interstitial pneumonia (formerly Hamman-Rich syndrome), and non specific interstitial pneumonia (NSIP) [4]. In 2002, an international multidisciplinary consensus classification of idiopathic interstitial pneumonias was proposed by the American Thoracic Society (ATS)/European Respiratory Society (ERS) [5]. This classification defined a set of histologic pattern that provided the basis for clinico-radiologic-pathologic diagnosis, with the final pathologic diagnosis being made after careful correlation with clinical and radiologic features. However, as discussed in several reports, the classification schemes of adult ILD are not satisfactory for the pediatric cases which seem to comprise a broader spectrum of disorders with a more variable clinical course [6]. In addition, pediatric histologic patterns often do not resemble pathologic features of lung tissues from adults and some forms are only observed in children younger than 2 years.

Among the proposed classifications for pediatric ILD, one strategy frequently used is to separate the primary pulmonary disorders and the systemic disorders with pulmonary involvement. Recently, an additional group has been introduced which is based on the concept that some pediatric ILD are observed more frequently in infants, while others are more specific to older children. The last ERS monography on ILD provided a chapter on pediatric classification which is based on a clear distinction between children aged 0-2 years and children over 2 years-old [7]. Indeed the stage of lung development and maturation should be taken into consideration when approaching a diagnosis of pediatric ILD. In this view, a new term "diffuse lung disease" has recently been introduced that comprises a diverse spectrum of lung disorders with impaired gas exchange and diffuse infiltrates by imaging. These disorders, more prevalent in young children, include diffuse developmental disorders, lung growth abnormalities, neuroendocrine cell hyperplasia and pulmonary interstitial glycogenosis, surfactant dysfunction disorders, disorders related to systemic diseases, disorders of immunocompromised host, and disorders of normal host caused by various insults such as aspiration syndrome or infections [8]. Some diseases are mostly observed in older children such as systemic diseases, idiopathic disorders as described in adults (DIP, UIP, NSIP and lymphoid interstitial pneumonia (LIP)), unclassifiable ILD and also infectious disorders [9].

It is important to point out that the pathologic processes underlying the so-called diffuse lung diseases involve not only the alveolar structure but also the distal part of the small airways and the conducting zone, *i.e*. the terminal bronchioles. Terminal bronchioles are lined with a simple cuboidal epithelium containing Clara cells, basal cells and a limited number of ciliated cells. Clara cells secrete nonsticky proteinaceous compounds to maintain the airway in the smallest bronchioles, which constitute the quiet zone between the conducting and the respiratory lung zones [10]. The terminal bronchioles are surrounded by a spiral of smooth muscle. Each of the terminal bronchioles divides to form respiratory bronchioles which contain a small number of alveoli. Consequently, the term of diffuse lung disease refers to disorders that can affect both the distal part of the conducting and the respiratory lung zones, and include ILD as well as pathological processes leading to obstruction/obliteration of small airways [8]. Therefore, diffuse lung diseases encompass a broader group of diseases than ILD which refers to disorders that affect the respiratory function of the lung and consequently the pulmonary structure responsible of the diffusion of gases between blood and air (i.e. the alveolar epithelium, the interstitium, and the pulmonary capillary endothelium).

The present review focuses on ILD in immunocompetent children, and excludes pulmonary consequences of previous lung injury in situations of chronic aspiration syndromes, resolving acute respiratory distress syndrome, and bronchopulmonary dysplasia.

## Epidemiology

An estimated prevalence of 3.6 per million has been reported by Dinwiddie and coworkers through a national survey of chronic ILD in immunocompetent children in the United Kingdom and Ireland over a three year period (1995-1998) [1]. This prevalence is certainly under-estimated due to the lack of standardized definitions and the absence of organized reporting systems. From the limited published data composed mainly of case reports and small series, it seems that pediatric ILD occurs more frequently in the younger age and in boys [11]. In addition, nearly 10% of cases appear to be familial [12].

## Pathophysiology

### Critical role of the alveolar epithelium

The understanding of the mechanisms underlying the development and progression of ILD remains elusive [13,14]. Indeed, for a long time, chronic ILD and pulmonary fibrosis were believed to result mainly from chronic inflammation following an initial injury to the alveolar epithelial lining [15,16]. In cases of limited injury, it was thought that the reparative attempt could reverse the trend toward fibrosis. By contrast, in situations of continuing injury, the repair process driven by inflammatory molecules produced by the local cells will result in scarring and structural changes. Therefore, by targeting the inflammatory response, the belief was that fibrosis could be prevented or controlled. This theory explains the large use of anti-inflammatory therapy with, however, limited clinical efficacy.

Based on clinical and experimental observations, a new paradigm has progressively emerged with the alveolar epithelium being viewed as a key actor in the development of ILD [17-19]. Following injury, alveolar epithelial cells (AEC) may actively participate in the restoration of a normal alveolar architecture through a coordinated process of re-epithelialization, or in the development of fibrosis through a process known as epithelial-mesenchymal transition (EMT) [20]. Complex networks orchestrate EMT leading to changes in cell architecture and behaviour, loss of epithelial characteristics and gain of mesenchymal properties. The reasons for epithelial cell loss and inappropriate re-epithelialisation are still debated, but ongoing apoptosis is believed to be a key component in the progression of the disorder [21]. Prolonged denudation of the basement membrane contributes to altered interactions and cross-talk between AECs and mesenchymal cells, resulting in profound modifications of cell functions with imbalanced production of oxidants, proteases, and polypeptide mediators including cytokines and growth factors such as Transforming Growth Factor (TGF)-β and Endothelin (ET)-1. A consequence is the perpetuation of a vicious cycle with TGF-β promoting epithelial cell apoptosis, which in turn increases the local production of TGF-β [22]. ET-1 is also considered to be an important actor, based on the current knowledge of its numerous functions including fibroblast and smooth muscle cell mitogen, and stimulant of collagen synthesis [23,24]. Recent studies showed that ET-1 is produced by AEC, and could induce alveolar EMT via stimulation of endogenous TGF-β production.

### Multiple causes and pathways

**Figure 1:**
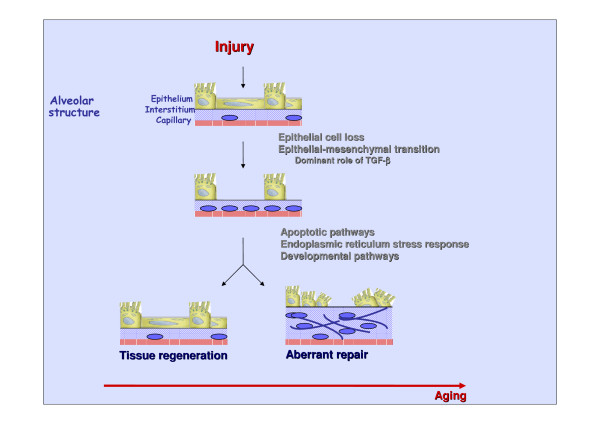
**Mechanisms and pathways involved in the response of the alveolar structure of the lung to injury**. Abbreviation: Transforming Growth Factor (TGF)-β.

ILD may be caused by myriad etiologies with differing prognoses and natural history. Indeed, multiple factors may injure the alveolar epithelium and initiate the development of ILD [25]. The initiating injury can be introduced through the airways and the circulation, or can occur as a result of sensitization. Consequently, the mechanisms underlying disease progression will be influenced by the causative event as well as by the host and the environment. These mechanisms are developed through interactions of multiple pathways, which include apoptotic pathways, developmental pathways, and endoplasmic reticulum (ER) associated pathways (Figure 1).

Apotosis plays a central role in lung remodeling associated with ILD [26]. An important molecule in the events associated with epithelial cell apoptosis is TGF-β, which is overexpressed in ILD. Downstream events linked to upregulation of TGF-β include modifications in the expression of various components of the cell cycle machinery, mainly the cyclin-dependent kinases (CDK) system that plays an essential role in ensuring proper cell cycle progression. Recently, much work has been focused on the protein p21cip1, a member of the CDK inhibitor family. This protein promotes cell cycle arrest to apoptosis in cases of cellular DNA damage. Interestingly, upregulation of p21cip1 has been reported in the lung tissues of patients with pulmonary fibrosis, primarily in hyperplastic alveolar epithelial cells [27] The increased expression of p21cip1 can favour the process of epithelial cell apoptosis. Apoptotic cells can also produce TGF-β. A consequence would be the perpetuation of a vicious cycle with TGF-β promoting epithelial cell apoptosis, which in turn increases the local production of TGF-β.

Recently, it has been suggested that genes associated with lung development and embryonic pathways could be involved in aberrant epithelium-mesenchymal crosstalk and epithelial plasticity, and could therefore participate in the development of chronic ILD. Selman and coworkers reported that lung fibrosis is characterized by enrichment for genes associated with cell adhesion, extracellular matrix, smooth muscle differentiations, and genes associated with lung development [28-31]. During EMT in the embryonic period, cells undergo a switch from a polarized epithelial phenotype to a highly motile mesenchymal phenotype [32]. Molecular processes governing EMT are induced by a cooperation of receptor tyrosine kinases or oncogenic Ras (RTK/Ras) pathway and TGF-β signaling [33]. Recently, additional pathways and effectors have been reported to play a role in the induction of EMT, such as Wnt//β-catenin, Notch and Sonic hedgehog signalling [34].

**Figure 2:**
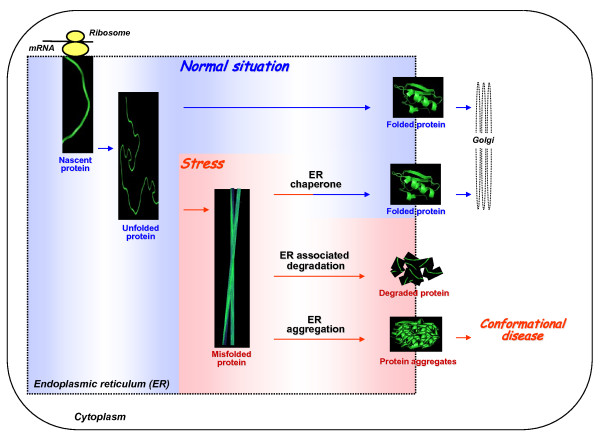
**Alveolar structure disorder-associated ILD and ER stress**. The (Endoplasmic Reticulum) ER and its protein maturation machinery allow the synthesis of mature secretory and membrane proteins with specific folded conformation. In situations of stress induced by genetic mutations or environmental factors, unfolded or misfolded proteins are retained in the ER and induce a defence mechanism called the ER stress response. The induction of ER chaperones is critical to increase the ER folding capacity allowing the production of correctly folded protein. When this defence mechanism is impaired, the misfolded proteins can either be degraded by the proteasome or form protein aggregates. Protein aggregates are toxic and can cause conformational diseases. Within the alveolar epithelium, misfolding of SP-C could trigger induction of intra-cellular aggregate formation and ER stress, with consequently development of alveolar structure disorder-associated ILD and conformational disease.

Recent reports strongly suggest that the ER stress may represent an important mechanism of the altered repair process observed in the alveolar epithelium of fibrotic lung [35]. Situations associated with abnormal regulation of the cascade of events leading to the formation of mature protein result in either misfolding or mistargeting of the protein. These events trigger induction of intracellular aggregate formation and ER stress, which can lead to cell death through apoptosis and autophagic pathways [36,37]. Several stimuli including oxidant-antioxidant imbalance, viral proteins, inflammatory molecules, nutrient deprivation may induce ER stress [38,39] (Figure 2). Among the cytoprotective mechanisms available are the ER chaperones such as binding immunoglobulin protein (BiP). Interestingly, mutant BiP mice have been reported to die within several hours of birth from respiratory failure due to impaired secretion of pulmonary surfactant by type 2 AEC. In these animals, expression of surfactant protein (SP)-C was reduced and the lamellar bodies were malformed, indicating that BiP plays a critical role in the biosynthesis of surfactant [40]. Several recent reports suggest the possible implication of ER stress in ILD, with activation of stress response markers in fibrotic lung tissues.

### Surfactant deficiency and stem cell dysfunction

It is now well established that surfactant dysfunction plays an important role in the development and progression of ILD. Pulmonary surfactant is a multimolecular complex constituted of phospholipids and proteins secreted by type 2 AEC into the alveolar space. It assures alveolar stability by reducing surface tension along the epithelial lining and this role involves mainly the lipids and the specific hydrophobic SP, SP-B and SP-C. Other important players in surfactant metabolism include the ATP-binding cassette, sub-family A, member 3 (ABCA3) and the thyroid transcription factor 1 (TTF-1).

Surfactant deficiency can be induced by a number of primitive and secondary mechanisms. Among them are genetic defects with mutations in SP-B gene (*SFTPB*) as well as genes coding for SP-C (*SFTPC*), ABCA3, and TTF-1 [41-43]. More than 30 *SFTPB *(located on chromosome 2) mutations have been identified among patients with a congenital deficiency in SP-B. For *SFTPC *located on chromosome 8, at least 35 mutations have been described, localized primarily in the COOH- terminal Brichos domain [44,45]. A proposed function of the Brichos domain is a chaperone-like activity, which could prevent misfolding and aggregation of the parent protein. Alterations in the Brichos domain could therefore lead to diseases through mechanisms related to abnormal protein processing and cell toxicity [46]. Recently, several studies have also documented the role of ABCA3 deficiency in ILD. ABCA3 functions in the transport of surfactant lipids into lamellar bodies and is required to maintain pulmonary surfactant phospho-lipid homeostasis. Another contributor of ILD is TTF-1 (NK2 homeobox 1) dysfunction. TTF-1 is a critical regulator of transcription for the surfactant protein SP-B and SP-C. It is encoded by a gene located on chromosome 14q13 and is composed of three exon and two introns [47]. It is expressed in the thyroid, brain and lung.

Stem cell dysfunction represents a new domain of investigation. Alveolar epithelium regeneration and repair requires activation and proliferation of tissue-resident (progenitor) cells and their differentiation to replace the damaged cells [48]. However, unlike cancer cells, stem cells are not immortal and display decreasing telomere length with aging [49]. Telomere shortening has been documented to be associated with reduced capacity for stem cell renewal, and decreased activity of telomerase, the polymerase responsible for telomere maintenance. The stem cells of the alveolar epithelium are the type 2 AEC, and expression of telomerase has been documented in these cells [48]. Experimental studies have also indicated that telomerase is expressed mainly during lung development with a peak expression before birth followed by a decrease to nearly undetectable levels in mature alveolar epithelium. Interestingly, telomerase expression and activity could be reinduced in normal quiescent type 2 AEC exposed to oxidative stress [50]. The current understanding is that a population of type 2 AEC may have the capacity to survive injury through telomerase activation, and consequently may be responsible for repopulation of the damaged alveolar epithelium. On the basis of reports of pulmonary disorders in dyskeratosis congenita (a rare hereditary disease of poor telomere maintenance), recent and exciting findings have documented mutations in the telomerase gene in familial idiopathic pulmonary fibrosis [51]. In addition, it is likely that environmental factors such as inflammation, oxidative stress, or virus infection may modify telomerase activity and account for the development of organ-specific disease associated with telomerase dysfunction. In this view, new data in chronic respiratory diseases support the concept that alveolar stem cell dysfunction may play an important role in the rate of progression or severity in ILD [52]. The question whether telomerase mutations or telomere dysfunction may be implicated in pediatric ILD needs to be addressed in prospective studies, one possible tool being determination of telomere length in circulating leukocytes.

### Role of age

The frequency of lung fibrotic disorders is much lower in children than in adults. Some clinical situations have features certainly unique to children, but many of these diseases overlap their adult counterparts with the primary event being injury and damage of the alveolar epithelium [11,13]. Yet, the overall outcome and prognosis of the diseases in children are thought to be less severe than in adult patients. In addition, pediatric ILD is more responsive to therapeutic strategies than adult ILD [9]. These differences may be explained by the types of initial injury, which may not be similar due to changes in the host environment. Another explanation is the modifications of the process of wound healing with age. Comparison of the response to injury in foetal and adult skin shows clear differences [53]. Skin wound healing in the foetus is characterized by complete regeneration of the skin and the absence of scar formation. Progressively with age, the skin looses the capacity to regenerate the original tissue architecture with the result being scar formation that extends outside the wound bed. The process of healing involves the coordinated regulation of cell proliferation and migration and tissue remodeling, predominantly by polypeptide growth factors [54]. The slowing of wound healing that occurs in the aged may be related to changes in the activity of these various regulatory factors. In a study on the role of aging in the development of cardiac fibrosis in the rabbit, differences in the cascade of events leading to myocardial remodeling were observed, with mainly the presence of more myofibroblasts synthesising collagen and expressing high levels of TGF-β in older animals [55]. A study of growth factors involved in skin wound healing in young and aged mice also showed age-dependent changes. Expression of all the fibroblast growth factors was diminished in aged mice, even in healthy skin. In addition, the post-wound regulation of expression of these factors and of TGF-β was less pronounced and slower than in young mice. These findings are in agreement with data observed in muscle that indicated significant alterations in the TGF-β production with age [56,57]. Other potential mechanism is linked to the observation that injury in adult tissues does in certain circumstances stimulate tissue regeneration, depending on the presence of small subsets of primitive stem cells. Stem cells are the self-renewing, primitive, undifferentiated, multipotent source of multiple cell lineages [49]. While such cells are critical for development and growth through childhood, residual pools of adult stem cells are hypothesized to be the source of the frequently limited tissue regeneration and repair that occurs in adults [58]. Unlike embryonic stem cells, adult stem cells are not immortal, and show decreasing telomere length with increasing age. The naturally limited replacement capacity of such endogenous stem cell pools may occur via exhaustion of the stem cell pool or arise as a consequence of inherited or acquired mutations that alter proper stem cell function [59]. The limited life span of cells may result from replicative senescence in response to various stresses including DNA damage, oxidants, and telomere erosion [52]. All these forms of injury have been documented in the lung from adult patients with ILD.

## Diagnosis of ILD

### Clinical presentation

The prevalence of children ILD is higher in the younger patients: more than 30% of patients are less than 2 years at diagnosis, as recorded by the recent ERS Task Force. 7% have parental consanguinity and nearly 10% of case siblings were affected by similar diseases. There is a male predominance with a sex ratio of 1.4. The presenting clinical manifestations are often subtle and non-specific. The onset of symptoms is, in most cases, insidious and many children may have had symptoms for years before the diagnosis of ILD is confirmed. However, the majority of patients has symptoms for less than one year at the time of initial evaluation. The clinical manifestations vary from asymptomatic presentation with radiological features suggestive of ILD to more characteristic presence of respiratory symptoms and signs such as cough, tachypnea and exercise intolerance [9,60]. These varying presentations are also reflected in the report published by Fan et al. who systematically evaluated the clinical symptoms and physical findings of 99 consecutive children with ILD [2]. Common symptoms at presentation included cough, dyspnea, tachypnea and chest wall retraction, exercise limitation and frequent respiratory infections. Cough is observed in almost 75% of the patients, is normally non-productive and does not disturb sleep. Tachypnea is observed in 80% of patients and is usually the earliest and most common respiratory symptom. Unexplained fever is also reported in almost one third of infants. Failure to thrive (37%), tiring during feeding and weight loss are also common symptoms, mainly in young patients. Although a history of wheezing may be elicited in almost 50% of the patients, wheezing is documented by physical examination in only 20% of the cases.

The frequent clinical findings are inspiratory crackles (44%), tachypnea and retraction. In a child with a normal birth history, these are strongly suggestive of ILD. Other findings associated with an advanced stage of lung disease include finger clubbing (13%) and cyanosis during exercise or at rest (28%) [9,61]. During physical examination it is essential to check the presence of associated non-respiratory symptoms such as joint disease, cutaneous rashes, and recurrent fever suggestive of collagen-vascular disorders. Details should also be obtained on precipiting factors such as feeding history, infections, or exposure to dust and drugs. In addition, information on relatives or siblings with similar lung conditions should be gathered.

### Chest imaging

Plain radiographs are usually performed in a child suspected of ILD at first presentation, but the information provided is often limited and the key chest imaging tool for diagnosis is the High Resolution Computed Tomography (HRCT), which can visualize the parenchymal structure to the level of the secondary pulmonary lobule.

HRCT technique for ILD diagnosis has been extensively discussed [62-64]. To optimise spatial resolution, there is a general agreement to use thin sections, the smallest field of view and a sharp resolution algorithm. The most common HRCT feature of ILD is widespread ground-glass attenuation. Intralobular lines, irregular interlobular septal thickening and honeycombing are less common findings. Large subpleural air cysts in the upper lobes adjacent to areas of ground-glass opacities have been also reported in young children with ILD. These cysts are interpreted as paraseptal or irregular emphysema. HRCT is useful for ILD diagnosis and selection of lung area to be biopsied. It is proposed that it also may contribute to monitor disease activity and/or severity. However, evaluation is still needed to support a role of HRCT as a follow up tool in pediatric patients.

### Pulmonary function testing

Pulmonary function testing (PFT) techniques are well established in children and adolescents. However, children aged 2-6 years represent a real challenge in pulmonary function assessment as they cannot be sedated and find it difficult to cooperate with all respiratory manoeuvres. In 2007, an ATS and ERS statement on PFT in preschool children summarized the current knowledge on the PFT techniques suitable for young children [65,66].

Although PFT does not provide specific information, it represents a useful investigation for both the diagnosis and the management of ILD [11]. Generally, in ILD, pulmonary function abnormalities reflect a restrictive ventilatory defect with reduced lung compliance and decreased lung volumes [67-69]. Vital capacity (VC) is variably diminished; the decrease in total lung capacity (TLC) in general is relatively less than in VC. Functional residual capacity (FRC) is also reduced but relatively less than VC and TLC, and residual volume (RV) is generally preserved; thus the ratios of FRC/TLC and RV/TLC are often increased. Airway involvement is observed only in a minority of patients. Lung diffusing capacity of carbon monoxide (DLCO) or transfer factor (TLCO) is often markedly reduced and may be abnormal before any radiological findings. However, DLCO corrected for lung volume may also be normal in many children. Hypoxemia as defined by a reduced resting arterial oxygen saturation (SaO_2_) or a reduced resting arterial oxygen tension is often present. Hypercarbia occurs only late in the disease course. During exercise the above described dysfunctions become even more pronounced. Thus, gas exchange during exercise might be a more consistent and sensitive indicator of early disease [3].

### Bronchoalveolar lavage

Bronchoalveolar lavage (BAL) usefully provides specimens for cytological examination, microbial cultures, and molecular analysis. Besides infections, BAL can be of diagnostic value in several situations. In the context of pulmonary alveolar proteinosis, BAL abnormalities are characterized by milky appearance fluid, abundant proteinaceous periodic acid schiff positive material, and presence of foamy alveolar macrophages (AM) [70]. BAL can also be diagnostic for pulmonary alveolar haemorrhage [11]. This diagnostic is easy when the BAL fluid has a bloody or pink color, but its gross appearance may be normal. Microscopic analysis may then be of value by documenting the presence of red blood cells in AM or haemosiderin laden AM [71]. Among other situations, the diagnosis of Langerhans cell histiocytosis can be performed with the use of the monoclonal antibodies revealing the presence of CD1a positive cells (in more than 5% of the BAL cells) [72]. Lipid disorders with lung involvement represent another indication of BAL. This includes congenital lipid-storage diseases (Gaucher's disease and Niemann-Pick disease) or chronic lipid pneumonia due to chronic aspiration [73,74]. However, in cases of aspiration syndromes, the presence of lipid laden AM is sensitive but not specific [75].

In other pathological situations, BAL can usefully serve to direct further investigations. Accumulation of BAL T-lymphocytes with prevalence of CD4+ cells is suggestive of sarcoidosis, whilst prevalence of CD8+ cells is suggestive of hypersensitivity pneumonitis [76]. Also, an increase in BAL eosinophils suggests pulmonary infiltrates associated with eosinophilia syndromes [77]. Depending of the underlying diseases, a number of cellular and molecular investigations can be proposed including the studies of various surfactant components, phospholipids and apoproteins [78].

### Tissue biopsies

With increasing recognition of the different patterns of ILD and their clinical significance, histological investigation has become increasingly important. Depending on disorder presentation, biopsy may concern more accessible organs than the lung such as the skin or the liver in sarcoidosis. Histological evaluation of lung tissue usually represents the final step in a series of diagnostic approaches.

Different methods may be used to obtain lung tissue. The major difference between individual methods lies mainly in balancing invasiveness against the potential for obtaining adequate and sufficient tissue for diagnosis. The techniques of choice are open lung biopsy and video assisted thoracoscopy biopsy. In children, open lung biopsy usually provides sufficient tissue with few complications related directly to the biopsy procedure [79]. Video assisted thoracoscopy biopsy is an alternative to open lung biopsy, and it has been shown that the procedure can be safely performed, even in small children [80]. The place of other methods like transbronchial lung biopsy and percutaneous needle lung biopsy in appropriate diagnosis of pediatric patients with ILD has to be established [81-83].

The lung histological patterns that can be observed in ILD have been reviewed by the ATS/ERS [5]. In children, they include mainly: DIP, NSIP, and LIP. DIP is characterized by airspaces filled with AM, thickened alveolar septa, scattered mixed inflammatory cells and minimal fibrosis. Many alveolar spaces are lined by hyperplastic type 2 AEC. Recently, association with surfactant disorders has been reported [41,84-86]. NSIP encompasses a broad spectrum of abnormalities with varying degrees of alveolar wall inflammation or fibrosis. The cellular pattern of NSIP is characterized by mild to moderate interstitial chronic inflammation and type 2 AEC hyperplasia in inflammation areas. It has been reported in a variety of underlying conditions including connective tissues diseases and surfactant disorders. LIP features include a marked diffuse infiltrate of mature lymphocytes, plasma cells and histiocytes within the pulmonary interstitium, particularly the alveolar walls. They are often associated with either connective tissues disorders or immunodeficiency states, both congenital and acquired [9]. Another pattern described mainly in adults is diffuse alveolar damage (DAD), which includes diffuse homogeneous thickening of alveolar interstitial walls with myofibroblast accumulation, prominent type 2 AEC hyperplasia and atypia, and hyaline membranes containing surfactant proteins and cellular debris [87]. Usual interstitial pneumonia (UIP) is rare in children [88]. It is characterized by severe remodeling of the alveolar structure with heterogeneous appearance consisting of contiguous areas of normal lung, dense scarring, and bronchiolar abnormal proliferation. Interstitial inflammation is usually mild to moderate. Histologic patterns of ILD unique to infancy are described below.

### Other tests

Laboratory tests are used to exclude a number of respiratory diseases in childhood that does not typically present with ILD such as chronic aspiration syndromes, resolving acute respiratory distress syndrome, tuberculosis, cystic fibrosis, bronchopulmonary dysplasia and diffuse pulmonary disease such as cystic fibrosis. Laboratory tests also verify the absence of immunodeficiencies [3].

When these conditions have been eliminated, the spectrum of investigations that should be performed for the diagnostic approach will be guided by the history and clinical presentation in each individual child. These investigations are discussed below for the various disorders. In addition, an increasing number of blood and BAL biomarkers for evaluation of disease severity and progression is currently investigated. The studied molecules include various cytokines and chemokines, surfactant protein D, Krebs von den Lungen-6 antigen (KL-6), matrix metalloproteinases MMP1 and MMP7 and defensins [89-92].

## Etiological diagnosis of ILD

A large number of pathological situations can impair gas exchange and contribute to progressive lung damage and ILD. Consequently, diagnosis approaches need to be organized by cause, with a clinical evaluation requiring a careful history paying attention to exposures and systemic diseases. Indeed, in a number of pathological situations, no final diagnosis is proposed and the conclusion reported by the physician in charge of the patient is ILD of unknown cause. However, information from recent studies highlights the concept that lung insults caused by substances from the environment or in the context of systemic diseases are largely under-estimated and should be more often discussed considered in the diagnostic process. Based on this consideration, the following diagnostic grouping for pediatric ILD can be considered 1) exposure-related ILD; 2) systemic disease-associated ILD; 3) alveolar structure disorder-associated ILD; and 4) ILD specific to infancy.

**Figure 3:**
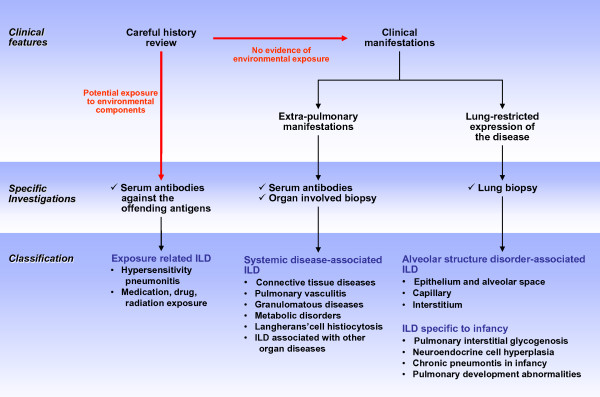
**Search for ILD etiology in children**. ILD is defined by the presence of diffuse infiltrates on chest radiographs or chest high resolution computed tomography, and abnormal pulmonary function tests with evidence of a restrictive ventilatory defect (in older children) and/or impaired gas exchange. The search for etiology requires a systematic step-by-step diagnostic strategy for identifying: exposure-related ILD; systemic disease-associated ILD; alveolar structure disorder-associated ILD; and ILD specific to infancy.

Accordingly, a step-by-step etiological diagnostic approach is required and is summarized in Figure 3. Once the diagnosis of ILD is established on clinical, radiological, and functional findings, a careful history should be obtained for potential exposure-related diseases leading to discuss the need for specific serum antibodies against offending antigens. The following step focuses on the search for systemic disease associated ILD, oriented by the presence of clinical and functional extra-pulmonary manifestations. In such situations, additional investigations should include specific serum antibodies and possibly tissue biopsies in organs other than the lung. Finally, elimination of these 2 groups of causes with a lung restricted expression of the disease allows discussing the potential interest of a lung biopsy.

### Exposure-related ILD

Exposure-related disease refers to diseases caused by a sufficient level of exposure (dose) to components with target organ contact, and subsequent biologic changes and clinical expression. Many agents have been associated with pulmonary complications of various types including ILD. The adult literature has provided extensive lists of candidate molecules [93]. In children, the potential involvement of these molecules is not similar as the environmental conditions and the use of therapeutic drugs differ. It is important to point out that exposure-related diseases are certainly under-estimated in the pediatric age. One reason is linked to the fact that the diagnosis is less often discussed than in adults as pediatricians and other child health care providers do not usually have the expertise necessary to take an environmental history. In this review, the most frequent causes of exposure-related ILD are discussed.

#### Hypersensitivity pneumonitis

Hypersensitivity pneumonitis (HP) is a cell-mediated immune reaction to inhaled antigens in susceptible persons [94,95]. In children, HP is often associated with exposure to antigens in the home environment as well as with certain hobbies. The most frequent types of HP include bird fancier's diseases, humidifier lung diseases, and chemical lung diseases. Bird fancier's diseases are induced by exposure to birds with the antigens being glycoproteins in avian droppings, and on feathers. Importantly, respiratory symptoms in exposed patients who have only one pet bird at home should raise the suspicion of HP [96]. Humidifier lung diseases (air conditioner lung, misting fountain lung, basement lung diseases) are caused mainly by free-living amoeba and nematodes, as well as bacteria and fungi. Chemical lung diseases can be induced by various inorganic antigens such as those from vaporized paints and plastics. Low-molecular-weight chemicals may react with proteins in the airways, thus forming complete antigens. Once exposure history is obtained, additional information is required and includes biologic tests allowing measurements of environmental contaminants and interpretation of the results by environmental medicine experts.

As HP is believed to be an adult disease, children are often diagnosed at the chronic stage of the disease resulting of a long-term exposure to low levels of inhaled antigens. Children can develop subtle interstitial inflammatory reactions in the lung without noticeable symptoms for months [97]. Clinical features in the classic form include non productive cough, dyspnea, malaise, asthenia and occasional cyanosis [95]. Lung function abnormalities are not specific and appear similar to changes observed in other ILD. HRCT abnormalities vary from ground glass attenuation predominantly in the mid-upper zone to nodular opacities with signs of air-trapping [62,63,98]. Laboratory tests focus mainly on the search for serum-precipitating IgG antibodies against the offending antigen [95]. However, the presence of these antibodies is considered to be of questionable clinical relevance for diagnosis, as it is observed in up to 50% of serum samples of exposed but asymptomatic individuals. BAL cell profile study typically shows an increase in total cell count with a remarkable elevation in the percentage of lymphocytes often over 50% with a decreased CD4/CD8 ratio [95,97]. However, in contrast to studies in adults, the CD4/CD8 ratio could be within the normal range for children [76]. Histopathologic evaluation of lung tissue is usually not necessary for the diagnosis of HP.

At the present time, there is no diagnostic test that is pathognomonic for HP, and only significant predictors of HP are identified. The most significant diagnostic tool is a detailed environmental exposure history. Other diagnostic features include: positive precipitating antibodies to the offending antigen; recurrent episodes of symptoms; symptoms occurring 4-8 h after exposure; occurrence of diffuse parenchymal lung disease by lung function and HRCT; BAL abnormalities with lymphocytic alveolitis and increased CD8+ T cells.

#### Medication, drug, radiation and tobacco exposure

Drugs used in inflammatory or cancer pediatric diseases can cause ILD. They include anti-inflammatory agents (e.g. aspirin, etanercept), immunosuppressive and chemotherapeutic agents (e.g. azathioprine, methotrexate, cyclophosphamide), antibiotics, cardiovascular agents, and, for teenagers, illicit drugs [99,100]. There are no distinct clinical, radiographic or pathologic patterns, and the diagnosis is usually made when a patient is exposed to medication known to result in lung disease, with a timing of exposure appropriate for disease development and elimination of other causes of ILD. Treatment relies on avoidance of further exposure and corticosteroids in markedly impaired patients.

Exposure to therapeutic radiation in the management of pediatric cancer may also results in ILD. Patients presenting within 6 months of therapy generally have radiographic abnormalities with ground glass patterns in both radiation-exposed and unexposed tissue [101].

The association between tobacco use and ILD is less well appreciated than the relation with chronic obstructive pulmonary disease (COPD). In addition, pediatric patients do not usually have a significant smoking history to develop respiratory disorders [102].

### Systemic disease-associated ILD

#### Connective tissues disease

Connective tissues disorders (CTD) are a heterogeneous group of immunologically mediated inflammatory diseases. Their origins are multifactorial with genetic, constitutional and environmental elements contributing to their development. CTD refers to any disease that has the connective tissues of the body as a primary target of pathology. The connectives tissues are composed of two major structural proteins, elastin and collagen, with different types of collagen proteins in each tissue [103]. Many CTD feature abnormal immune system activity associated with inflammation. Pulmonary manifestations of CTD may include both vascular and interstitial components. From recent reports, the incidence of ILD in the context of CTD appears to be higher than previously appreciated [104,105]. Importantly, ILD may precede the development of clinically obvious CTD, sometimes by months or years. Table 1 provides information on suggestive clinical and serological features in selected conditions. The main disorders to be considered in childhood are rheumatoid arthritis, systemic sclerosis, and systemic lupus erythematosus. The other include Sjögren syndrome, dermatomyositis and polymyositis, ankylosing spondylitis, and mixed connective tissue disease.

**Table 1:** Systemic disease-associated interstitial lung diseases: suggested clinical features and serotypes

CONNESTIVE TISSUE DISEASE	MAIN CLINICAL FEATURES	MAIN SEROLOGICAL FEATURES	HLA PREDISPOSITION
**Rheumatoid arthritis**	Arthralgia	RF IgM and IgA Anti-CCP Anti-keratin	HLA-DR SE
**Systemic sclerosis**	Sclerodactyly	ANA-SSc Anti-centromere Anti-topoisomerase I (Scl70) and II Anti-RNA polymerase	HLA-DR3, HLA-DPB1
**Systemic lupus** **erythematosus**	Skin rash Arthralgia Glomerulonephritis	ANA Anti-native DNA Anti-nucleosome Anti-Sm, RNP, SSA, SSB Anti-ribosome CIC	
**Sjögren syndrome**	Xerostomia Serophtalmia	ANA Anti-SSA, SSB RF Anti-RNP	
**Dermatomyositis** **and polymyositis**	Muscle weakness Skin rash	ANA Anti-Jo1 Anti-Mi2 Anti-SRP C-ADM-140	
**Ankylosing** **Spondylitis**	Bony ankylosis		HLA-B27
**Mixed connective** **tissue disease**	Raynaud phenomenon	Anti-U1-RNP	

**PULMONARY** **VASCULITIS**	**MAIN CLINICAL** **FEATURES**	**MAIN SEROLOGICAL** **FEATURES**	**HLA ** **PREDISPOSITION**

**Wegener's** **granulomatosis**	Glomerulonephritis Sinusitis	c-ANCA PR3	
**Churg-Strauss** **syndrome**	Asthma	p-ANCA	
**Microscopic** **polyangitis**	Glomerulonephritis Sinusitis Skin involvement	p-ANCA	
**Goodpasture** **syndrome**	Glomerulonephritis	anti-GMB	HLA-DRB1*1501
**Henoch-Schönlein** **purpura**	Purpura Glomerulonephritis	IgA deposition	HLA-DRB1
**Cryoglobulinemic** **vasculitis**	Skin involvment Hepatitis Glomerulonephritis	Cryoglobulin	

***Abbreviations*: **Rheumatoid factor (RF), Immunoglobulin (Ig), Human leucocyte Antigen (HLA), Anticyclic citrullinated peptide (anti-CCP), Antinuclear antibodies (ANA), Systemic sclerosis (SSc), Smith (Sm), ribonucleoprotein (RNP), circulating immune complex (CIC), anti-histidyl-t-RNA synthetase (Jo1), signal recognition particle (SRP), anti-U1-ribonucleoprotein antibody (anti-U1-RNP Ab); Cytoplasmic-staining (c) or Perinuclear-staining (p) anti-neutrophil cytoplasmic antibody (ANCA), anti-glomerular basement membrane (anti-GBM)

##### Rheumatoid arthritis

Rheumatoid arthritis (RA) is an inflammatory disorder defined by its characteristic di-arthroidal joint involvement. It is the most common CTD in children, but pulmonary involvement is less frequent than in adults. Genetic and environmental factors seem to be important contributors of disease progression, with influence of sex (more frequent in male), presence of two copies of the HLA-DRB1 "shared epitope" (HLA-DR SE) and anticyclic citrullinated peptide antibody (anti-CCP), and possibly tobacco exposure [106,107].Almost 50% of patients with RA have specific serologic abnormalities several years before the onset of joint symptoms, and the findings of elevated serum levels of IgM rheumatoid factor or anti-CCP is associated with a high risk for the development of RA [107].

##### Systemic sclerosis

Systemic sclerosis (SSc) is characterized by a progressive dermatologic abnormality [108]. Its etiology remains unknown; it is believed to be a complex disease in which interactions between environmental, auto-immune, and genetic factors result in various disease phenotypes [109]. Although it is a rare disease in childhood, the diagnosis is based on skin disease. Cardiopulmonary complications are common and have been associated with death in young patients. Almost all patients with SSc have serum antinuclear antibodies. The other autoantibody markers are listed in table 1. Recently, the presence of anti-DNA topoisomerase II autoantibody has been reported to be a key factor in the development of ILD, in association with class II MHC status (HLA-DR3, HLA-DPBI) [110].

##### Systemic lupus erythematosus

Systemic lupus erythematosus (SLE) is an auto-immune disorder characterized by the involvement and dysfunction of multiple organ systems. The mechanisms of tissue injury involve autoantibody production and immunocomplex formation leading to an inflammatory process. Diverse clinical phenotypes are observed, including a variety of mucocutaneous lesions, non erosive arthropathy, renal disease (glomerulonephritis and interstitial nephritis), lung disease, pericarditis, and a spectrum of neurologic disorders. Laboratory abnormalities are characterized by the presence of antibodies reactive to nuclear (ANA) and cytoplasmic antigens.

#### Pulmonary vasculitis

Pulmonary vasculitis are observed in vasculitic syndromes that preferentially affect small vessels (arterioles, venules, and capillaries). They include the anti-neutrophil cytoplasmic antibody (ANCA)-associated vasculitis (Wegener's granulomatosis, Churg-Strauss syndrome, and microscopic polyangitis) that share histologic similarities without immune deposits; anti-glomerular basement membrane (GBM) disease; Henoch-Schönlein purpura and cryoglobulinemia vasculitis. Vasculitic syndromes that affect large/medium vessels (such as Kawasaki's disease, polyarteritis nodosa) only occasionally affect the lung [111].

##### Wegener's granulomatosis

Wegener's granulomatosis (WG) is a rare disease of uncertain cause. It seems to affect children as much as adults with an increasing reported incidence around 2.75 cases/million/year, mostly in teenagers with a reported median age of 14.2 years (4-17 years) [112,113]. It is characterized by inflammation in a variety of tissues including blood vessels (vasculitis). WG primarily affects the upper respiratory tract, lung, and kidneys. The diagnosis is based on the combination of symptoms and a biopsy of affected tissue with necrotising granulomatous vasculitis in the absence of an infectious etiology. The diagnosis is further supported by positive blood tests for cytoplasmic-staining (c)-ANCA PR3 type [114].

##### Churg-Strauss syndrome

Churg-Strauss syndrome (CSS) is a granulomatous small-vessel vasculitis. The cause of this allergic angiitis and granulomatosis is not known, but autoimmunity is evident with the presence of hypergammaglobulinemia, increased levels of immunoglobulin E (IgE), and perinuclear-staining (p)-ANCA. The diagnosis relies on biopsy evidence for vasculitis and at least 4 criteria among the following: moderate to severe asthma, blood eosinophilia (at least 10%), and nonfixed pulmonary infiltrates with extravascular eosinophils on biopsy [115]. Twenty-nine pediatric cases have been reported so far in the literature, with lung involvement in 72% of [116].

##### Anti-glomerular basement membrane disease

Goodpasture syndrome is a rare disease that involves rapidly progressive kidney failure along with lung disease and is characterized by the deposition of anti-GBM antibodies. Several cases have been reported in the pediatric literature. The autoantibodies mediate tissue injury by binding to their reactive epitopes in the basement membranes. This binding can be visualized as the linear deposition of immunoglobulin along the glomerular basement membrane. The principle component of the basement membrane is type IV collagen which can be expressed as 6 different chains, from alpha1 to alpha6. The Goodpasture antigen has been localized to the carboxyl terminus of the noncollagenous domain of the alpha3 chain of type IV collagen. The anti-GBM antibody can usually be found in serum [117]. Strong evidence exists that genetics play an important role. Patients with Goodpasture disease have an increased incidence of HLA-DRB1 compared to control populations [118].

#### Granulomatous diseases

Granulomatous disorders are characterized by the presence of granulomas defined as a focal, compact collection of inflammatory cells in which mononuclear cells predominate. Granulomas form as a result of tissue injury by a wide variety of agents including micro-organisms, antigens, chemical, drugs and other irritants. In other situations including sarcoidosis, the etiologic factors remain to be determined.

##### Sarcoidosis

Sarcoidosis is a chronic inflammatory disease in which granulomatous lesions can develop in many organs, mainly the lung. Its cause remains obscure, and most likely involves environmental and host factors [119]. The current concept is that a still unknown stimulus activates quiescent T cells and macrophages leading to recruitment and activation of mononuclear cells, with, as a consequence, granuloma formation, alveolitis, and in some cases interstitial lung fibrosis [120]. Sarcoidosis is relatively uncommon among children. Its diagnosis is based on a combination of suggestive clinical features, with histologically-documented noncaseating granuloma, in the absence of other known causes of granuloma formation [121].

The incidence and prevalence of sarcoidosis are reported to be influenced by age, race and geographic localization [122]. Although the youngest patients reported were infants 2 and 3-months old, most of the cases in children occur in preadolescents and adolescents. From the national patient registry on patients with sarcoidosis in Denmark during the period 1979-1994, 81 patients with a confirmed diagnosis were ≤16 years of age [123]. The calculated incidence was 0.29 per 100.000 person-years. In children ≤4 years of age, the incidence was 0.06; it increased gradually to 1.02 in children aged 14-15 years. Marked racial differences in the incidence and prevalence of sarcoidosis have been reported by many authors [122]. Various reports in the literature also indicate that race and ethnicity affect both the patterns of organ involvement and disease severity. In a follow-up study we have conducted in 21 children with pulmonary sarcoidosis, 12 children were Black [124]. Also the number of organs involved was higher in the Black than in the Caucasian children.

Clinical manifestations in sarcoidosis are the consequences of local tissue infiltration with sarcoid granuloma. Therefore, disease expression depends on the organ or system involved and a variety of symptoms and physical findings can be observed [125]. The modes of presentation include non-specific constitutional symptoms, alone or associated with symptoms related to specific organ involvement. In the report of children with sarcoidosis in Denmark, the most common non specific symptoms were asthenia, weight loss, and fever [123]. Clinical findings mainly include respiratory manifestations, lymphadenopathy, skin lesions, ocular and central nervous system abnormalities. The most common radiographic findings are hilar lymph node enlargements, with or without lung changes. Lung function abnormalities are frequently observed in children with restrictive pulmonary pattern and abnormal diffusing capacity [126]. Other investigations such as BAL documenting a lymphocytic alveolitis with increased CD4/CD8 ratio, and elevated serum angiotensin-converting enzyme may provide additional evidence of sarcoidosis [127].

##### Other granulomatous disorders in children

A number of pathological situations are associated with granulomatous disorders defined by the presence of non-caseating granuloma in biopsied tissues. Infections are the main causes of other granulomatous diseases, and are in some cases related to disorders of neutrophil function such as chronic granulomatous disease (CGD) [128]. Most children with CGD present with recurrent bacterial and fungal infections. The most frequently encountered pathogens are *Staphylococcus aureus*, *Aspergillus*, *Burkholderia cepacia*, and enteric gram negative bacteria [129]. The most prominent pulmonary lesions include an extensive infiltration of the lung parenchyma and hilar adenopathy. In some situations, a homogeneous distribution of small granulomatous lesions can occur, with a radiological appearance of miliary tuberculosis.

The other granulomatous diseases can be seen in other described diseases, such as immune disorders (including Crohn's disease and histiocytosis X), HS pneumonitis, vasculitis disorders or neoplasms.

#### Metabolic disorders

##### Lysosomal diseases

Gaucher's disease is an autosomal recessive disease and the most common of the lysosomal storage diseases. It is caused by a genetic deficency of the enzyme lysosomal gluco-cerebrosidase that catalyses the breakdown of glucocerebroside, a cell membrane constituent of red and white blood cells. The consequence is an accumulation of glucocerebroside in reticuloendothelial cells, leading to excessive deposition of fatty material in the spleen, liver, kidneys, lung, brain and bone marrow. Pulmonary expression is mainly characterized by physiologic involvement (reduction in lung the diffusion capacity and the functional residual volume). Lung imaging may show interstitial changes [130].

Niemann-Pick diseases are genetic diseases primarily due to deficiency of sphingomyelinase resulting in the accumulation of sphingomyelin within lysosomes in the macrophage-monocyte phagocyte system, mainly the brain, spleen, liver, lung, and bone marrow. Histology demonstrates lipid laden macrophages in the marrow, as well as "sea-blue histiocytes" on pathology. The infantile form with a dominant neurologic expression is rapidly fatal. In older patients, cases of ILD have been reported [131].

Hermansky-Pudlak syndrome is a heterogeneous group of autosomal recessive disorders associated with accumulation of a ceroid-like substance in lysosomes of a variety of tissues. It is characterized by albinism, bleeding tendency associated to poor platelet aggregation and systemic complications associated to lysosomal dysfunction. A chronic inflammatory process may explain the progressive development of ILD and fibrosis [132].

##### Familial hypercalcemia with hypocalciuria

Familial hypercalcemia with hypocalciuria is caused by autosomal dominant loss-of-function mutations in the gene encoding the calcium-sensing receptor (CASR), a G-protein coupled membrane receptor expressed in many tissues [133]. Loss-of-function mutations in CASR impair the feedback inhibition of parathyroid hormone secretion in response to a rise in the blood calcium concentration. The result is hypercalcemia associated with inappropriately normal or mildly elevated levels of parathyroid hormone. In the kidneys, mutations in CASR prevent the feedback inhibition of calcium reabsorption in situation of hypercalcemia, leading to relative hypocalciuria. Respiratory symptoms are usually mild and associated with reduction in the lung diffusion capacity. Lung histology indicates the presence of foreign body giant cells and mononuclear cells infiltrating the alveolar interstitium, without circumscribed granulomas.

#### Langerhans'-cell histiocytosis

Langerhans'-cell histiocytosis is part of the histiocytosis syndromes, which are characterized by an abnormal proliferation of Langerhans' cells [134]. The Langerhans cells are differentiated cells of monocyte-macrophage lineage that function as antigen-presenting cells. The origin of the expanded population of Langerhans' cells is unknown; in adults, the only consistent epidemiologic association is with cigarette smoking. These cells may form tumors, which may affect various parts of the body. Most cases of pediatric Langerhans'-cell histiocytosis are observed in children between ages 1 and 15 years, with usually bone involvement (80%) including the skull. The tumors produce a punched-out appearance on bone X-ray, and can cause fracture without apparent traumatism. Langerhans'-cell histiocytosis can also affects various organs including the lung [135].

Children with pulmonary Langerhans'-cell histiocytosis present in a variety of ways. They can be asymptomatic or present common symptoms such as nonproductive cough and dyspnea. HRCT of the chest is a useful and sensitive tool for the diagnosis. Indeed, the combination of diffuse, irregularly shaped cystic spaces with small peribronchiolar nodular opacities, predominantly in the middle and upper lobe, is highly suggestive of pulmonary Langerhans'-cell histiocytosis [63]. Other abnormalities include ground-glass attenuation. The presence of increased numbers of Langerhans' cells in BAL fluid (identified by staining with antibodies against CD1a) with a proportion greater than 5 percent is also strongly suggestive of pulmonary Langerhans'-cell histiocytosis. Histologically, the cellular lesions forms nodules containing a mixed population of cells with variable numbers of Langerhans' cells, eosinophils, lymphocytes, plasma cells, fibroblasts, and pigmented alveolar macrophages.

#### ILD associated with other organ diseases

Several forms of ILD have been reported to occur with inflammatory bowel diseases (Crohn's disease) and celiac disease [136]. Primary biliary cirrhosis and chronic hepatitis have also been reported to be associated with parenchymal lung dysfunction [137,138]. In addition, there are reports on ILD in association with neurocutaneous disorders (tuberous sclerosis, neurofibromatosis, ataxia-telangiectasia) and amyloidosis [139].

### Alveolar structure disorder-associated ILD

**Figure 4:**
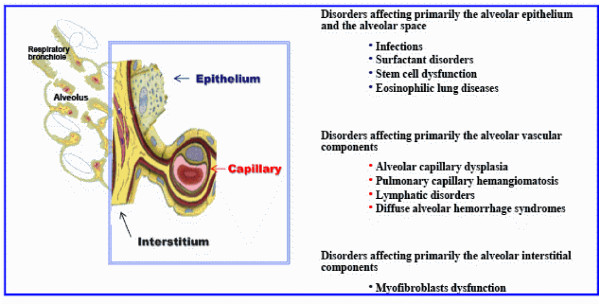
**Alveolar structure disorder-associated ILD**. Depending on the causes, the alveolar structure components can be involved differently and serve as primary targets of the underlying pathological processes. Based on history, clinical presentation, BAL and lung tissue information, the disorders can be gathered in groups according to the predominant alveolar targets: epithelium, vascular or interstitial components.

Depending on the causes, the components of the alveolar structure (the epithelium and the alveolar space, the interstitium, and the pulmonary capillary endothelium) can be involved differently and can serve as primary targets of the underlying pathological processes. Based on history, clinical presentation, BAL data, and, most important, on information from lung tissue studies, the disorders can be gathered in groups according to predominant structural targets (Figure 4).

#### Disorders affecting primarily the alveolar epithelium and the alveolar space

The disorders affecting primarily the alveolar epithelium and the alveolar space share common histopathological description, with preserved pulmonary architecture, hyperplasia of type 2 AEC, interstitial infiltrates composed of immuno/inflammatory cells and scattered myofibroblasts, and the alveolar space filled with either immuno/inflammatory cells, desquamated materials, or components derived from surfactant lipid and protein complex. In the coming years, it is likely that the list of disorders will expand rapidly with the availability of specific tissue markers. Currently, the following grouping can be proposed: infections, surfactant disorders, and eosinophilic lung diseases.

##### Infections

The role of infection, mainly viral, in the development and progression of ILD is sustained by a number of human and experimental reports. From recent knowledge, it is strongly suggested that latent viral infections may be involved in the pathogenesis of ILD, through targeting of the alveolar epithelium. The main virus implicated include adenovirus, members of human herpes virus family (Epstein-Barrr virus and cytomegalovirus), and respiratory syncitial virus [140]. Number of other viruses can also be involved such as Influenza A, hepatitis C, or even Human Immunodeficiency Virus (HIV) in immunocompetent children [141-144].

Human adenovirus being predominantly respiratory pathogens, adenovirus infections can cause a variety of pulmonary symptoms and can persist for long periods of time. Several studies in adult patients have indicated that the adenovirus gene product E1A could be detected in lung tissues by in situ hybridization in up to 16% of cases of idiopathic pulmonary fibrosis. The causative role of the virus in the initiation of the disease remains uncertain, but it may be an important factor in its progression as treatment with corticosteroids may make patients more susceptible to adenovirus infection or reactivation from latency. E1A has been shown to increase the production of TGF-β and to induce lung epithelial cells to express mesenchymal markers, thereby contributing to remodeling of the alveolar structure [145]. Isolation of the virus from the throat and serologic studies are diagnostic supportive, but the diagnosis is confirmed by the detection of the virus in lung tissues.

Epstein-Barrr virus (EBV) and cytomegalovirus (CMV) are widespread pathogens that share the characteristic ability of herpesviruses to remain latent within the body over long periods. In mice, the control of herpesviruses replication have also been reported to be associated with the arrest of lung fibrosis [146]. EBV is present in all populations, infecting more than 95% of individuals within the first decades of life. Infection by CMV is reported in 60% of individuals aged 6 and older and more than 90% of aged individuals have antibodies against CMV. In addition, CMV is also the virus most frequently transmitted to a developing fetus. Most healthy people who are infected by EBV and CMV after birth have no symptoms, but infection is important to certain high-risk groups of infants and immunocompromised individuals. Several studies in the adult literature have reported an increased incidence of EBV and CMV infection in patients with pulmonary fibrosis, associated with virus DNA-positive lung tissue biopsies in several cases [147]. However, so far, no evidence of causal relationship between viruses and pulmonary fibrosis has been provided.

Respiratory syncytial virus (RSV) is the most common cause of viral lower respiratory tract infection. It affects people of all ages, and can cause severe disease in infants, in older immunodeficient children and the elderly. An intriguing feature of RSV infection is the susceptibility of previously infected individuals to reinfection with antigenically closely related viruses or the identical virus strain. Recently, increased interest has been focused on the contribution of persistent RSV in several chronic lung diseases including chronic obstructive pulmonary disease [148]. The role of RSV in the physiopathology of theses disorders as well as and the mechanisms of its persistence remain to be elucidated [149]. Interestingly, in a recent work on the histopathology of untreated human RSV infection, the presence of the virus in AEC has been documented [150]. From these various data, a role of RSV in the development of ILD needs to be investigated. Immunostaining with RSV-specific antibodies of tissues from lung biopsy should be proposed.

Among the other pathogens, C*hlamydophila pneumoniae *and *Mycoplasma pneumoniae *are currently drawing increasing consideration. They are frequent causes of community acquired pneumonia in children. Before the age of 10 years, almost 70% of children have had C*hlamydophila pneumoniae *infection based on serological studies [151]. These pathogens are intracellular organisms that primarily infect respiratory epithelial cells and alveolar macrophages and have the propensity to persist within several cell types such as macrophages. They are well known to cause a wide variety of respiratory manifestations, with possible progression towards diffuse parenchymal diseases associated with interstitial infiltrates on chest imaging and reduction in the lung diffusion capacity [152]. Regarding *Legionella pneumophilia *infection, progression towards ILD has been infrequently reported in adult patients.

Results from recent studies provided evidence that viruses can infect the alveolar epithelium and may be documented in lung tissues from patients using virus DNA detection and immunohistochemistry. A number of specific antibodies are currently available and should prompt to investigate the presence of the above cited viruses in the lung tissues from children with ILD.

##### Surfactant disorders

Surfactant disorders include mainly genetic surfactant protein disorders and pulmonary alveolar proteinosis

The deficiency in SP-B is a rare autosomal recessive condition known to be responsible for lethal neonatal respiratory distress. Rare survivals have been described in partial deficiencies [153,154]. The *SFTPC *mutation I73T (c.218 T > C) is the more prevalent mutation. Others are described in only one family. The phenotype associated with *SFTPC *mutations is extremely heterogeneous leading from neonatal fatal respiratory failure to children and adults chronic respiratory disease with ILD [45]. Recessive mutations in the *ABCA3 *gene were first attributed to fatal respiratory failure in term neonates but are increasingly being recognized as a cause of ILD in older children and young adults. Over 100 *ABCA3 *mutations have been identified in neonates with respiratory failure and in older children with ILD [86,155-161]. Mutations in the *TTF-1 *gene are associated with "brain-lung-thyroid syndrome" which combines congenital hypothyroidism, neurological symptoms (hypotonia, chorea), and ILD of variable intensity [162-168]. So far, few mutations have been reported, mostly in exon 3 [169,170].

Pulmonary alveolar proteinosis (PAP) is a rare lung disorder characterized by alveolar filling with floccular material derived from surfactant phospholipids and protein components. PAP is described as primary or secondary to lung infections, hematologic malignancies, and inhalation of mineral dusts. Recently, the importance of granulocyte/macrophage colony-stimulating factor (GM-CSF) in the pathogenesis of PAP has been documented in experimental models and in humans. GM-CSF signaling is required for pulmonary alveolar macrophage catabolism of surfactant. In PAP, disruption of GM-CSF signaling has been shown, and is usually caused by neutralizing autoantibodies to GM-CSF. Therefore, the emerging concept is that PAP is an autoimmune disorder resulting in macrophage and neutrophil dysfunction. In a recent report, it has been reported that GM-CSF autoantibodies are normally present in healthy individuals, but at lower levels than in PAP patients [171]. In addition, in vitro experiments indicated that these autoantibodies reduce GM-CSP signaling similarly in healthy individuals and in PAP patients. At levels above a critical threshold, GM-CSF autoantibodies are associated with multiple impaired GM-CSF dependent myeloid function [172]. Several cases of genetic defects in the common beta chain for the GM-CSF receptor have been documented [173].

##### Eosinophilic lung diseases

Eosinophilic lung diseases constitute a diverse group of disorders of various origins. The diagnosis is suggested by the presence of pulmonary infiltrates on chest imaging and peripheral eosinophilia. It is confirmed by the presence of increased amounts of eosinophils in BAL and/or lung tissue eosinophilia. In this section, eosinophilic vasculitis will not be discussed (see chapter 6.2.2). The search for an etiology includes a combination of clinical and laboratory investigations. Eosinophilic lung diseases of known cause in children include mainly allergic bronchopulmonary aspergillosis, parasitic infections and drug reactions. Eosinophilic lung diseases of unknown cause comprise Loeffler syndrome (characterized by migrating pulmonary opacities), acute eosinophilic pneumonia, and chronic eosinophilic pneumonia [174,175]. The idiopathic hyper-eosinophilic syndrome is a rare disorder observed mainly in adults; it is characterized by prolonged eosinophilia and a multiorgan system dysfunction due to eosinophil infiltration with pulmonary involvement documented in almost half of the patients [176,177].

#### Disorders affecting primarily the alveolar vascular components

##### Alveolar capillary dysplasia and pulmonary capillary hemangiomatosis

The pulmonary capillaries form a dense sheet-like meshwork composed of short interconnected capillary segments. The capillary meshes are wrapped over the alveoli, with only a single sheet of capillaries between adjacent alveoli on the same alveolar duct. Impaired development of this vascular network can be caused by genetic defects, prematurity or injury. Aberrant angiogenesis documented in pediatric patients include mainly alveolar capillary dysplasia, and pulmonary capillary hemangiomatosis [178]. Alveolar capillary dysplasia is a rare disorder, presenting with persistent pulmonary hypertension of the newborn [179]. The strongest diagnostic features are poor capillary apposition and density, allied with medial arterial hypertrophy and misalignment of pulmonary vessels [180]. Pulmonary capillary hemangiomatosis is also a rare disease that is characterized by proliferation of capillary-sized vessels within the alveolar walls of the lung [181]. Intimal thickening and medial hypertrophy of the small muscular pulmonary arteries are present resulting in elevated pulmonary vascular resistance. Most cases appear sporadic. Chest imaging shows nodular pulmonary infiltrates and septal lines. A definitive diagnosis can be made only by histologic examination. Interestingly, capillary proliferation in the alveolar wall has been reported in hereditary haemorrhagic telangiectasia [182].

##### Lymphatic disorders

Alveolar structure formation is characterized by refinement of the gas exchange unit and functional adaptation of endothelial cells into vessels including pulmonary lymphatics. The pulmonary lymphatic network promotes efficient gas exchange through maintaining interstitial fluid balance. Lymphatic disorders can be classified as primary or secondary.

Congenital errors of lymphatic development can lead to primary pulmonary lymphatic disorders that include lymphangiomas and lymphangiomatosis, lymphangiectasis, and lymphatic dysplasia syndrome [183,184]. Lymphangiomas are focal proliferations of well differentiated lymphatic tissue, and lymphangiomatosis describes the presence of multiple lymphangiomas. Most of these disorders are discovered in fetuses or during the early postnatal period. Lymphangiectasis is characterized by pathologic dilation of lymphatics. The term "lymphatic dysplasia syndrome" includes congenital chylothorax, and the yellow nail syndrome (a triad of idiopathic pleural effusions, lymphedema, and dystrophic nails) [185]. Secondary forms of lymphatic disorders result from a variety of processes such as chronic airway inflammation that impair lymph drainage and increase lymph production [186].

##### Diffuse alveolar hemorrhage syndromes

Diffuse alveolar hemorrhage (DAH) syndromes are caused by the disruption of alveolar-capillary basement membrane as a consequence of injury to the alveolar septal capillaries, and less commonly to the arterioles and veinules. The hallmarks are intra-alveolar accumulation of red blood cells, fibrin, and hemosiderin-laden macrophages. It is important to point out that approximately one third of patients with DAH do not manifest hemoptysis, and BAL can be extremely helpful if this entity is suspected by showing the presence of siderophages or red blood cells within the alveoli. DAH can be observed in association with systemic findings or without evidence of associated diseases.

In children, situations of DAH in the context of other disorders are reported in several forms of vasculitis discussed above. Other disorders that can also be accompanied by DAH include pulmonary hypertension and congenital heart diseases, pulmonary veino-occlusive disease, arteriovenous malformations and hereditary haemorrhagic telangiectasia, coagulation disorders, and celiac disease [187].

In the absence of systemic findings, isolated pulmonary capillaritis should be discussed with the search for positivity of the antiglomerular basement membrane antibody with linear deposits in the lung tissue biopsy as well as suggestive serologic features such as p-ANCA antibodies [188].

Idiopathic pulmonary hemosiderosis is a diagnosis of exclusion based on patient presentation with acute, subacute, or recurrent DAH, on the results of lung biopsy showing evidence of 'bland' pulmonary hemorrhage (ie, without capillaritis or vasculitis), and after exclusion of the conditions listed above [189]. In this situation, red blood cells leak into the alveolar space without evidence of damage and/or inflammation of the alveolar capillaries. In addition, the diagnosis of idiopathic pulmonary hemosiderosis can only be considered after exclusion of diseases induced by environmental factors such as pesticide and cow's milk (Heiner's syndrome) [190]. This syndrome is a hypersensitivity disease that affects primarily infants, and is caused by antibodies to cow's milk proteins. The diagnosis is supported by positive milk precipitin test and rapid improvement of symptoms and pulmonary infiltrates on chest imaging after exclusion of milk proteins.

#### Disorders affecting primarily the alveolar interstitial components

In the resolution phase of tissue injury, elimination of mesenchymal cells and recruited inflammatory cells is essential for restoration of normal cellular homeostasis. Dysregulated repair process in ILD is associated with accumulation and dysfunction of interstitial fibroblasts [191]. In the coming years, it is likely that progress in the understanding of the mechanisms involved in the impaired myofibroblast apoptosis as well as evasion of these cells from immune surveillance will open new areas of investigations and will provide support for the characterization of disorders that affect primarily the alveolar interstitial components in pediatric ILD. Indeed, recently, distinct intrinsic differences in gene expression pathways has been reported between control and lung fibrosis myofibroblasts which suggests that ILD myofibroblasts are pathological cells with fundamental changes [192].

### ILD specific to infancy

In the context of ILD, pulmonary interstitial glycogenosis, neuroendocrine cell hyperplasia, and chronic pneumonitis in infancy have been reported to be exclusively observed in very young children [8].

Pulmonary interstitial glycogenosis (PIG) is a non lethal disease, reported in neonates with respiratory distress syndrome developed shortly after birth [193,194]. Very few cases are described so far but it seems to have a male preponderance [195]. The histological hallmark of pulmonary interstitial glycogenosis is the accumulation of monoparticulate glycogen in the interstitial cells on lung biopsy. It is thought to represent a maturation defect of interstitial cells that leads them to accumulate glycogen within their cytoplasm [8,196]. It is discussed that PIG could meet "chronic pneumonitis in infancy" as this remains a generalized term [87]. As well, PIG could be considered as a premature lung disease, but more than half of published cases were in term infants [195,197,198]. The long term consequences in these infants need to be ascertained.

Neuroendocrine cell hyperplasia of infancy (NCHI) is also a non lethal disease characterized by tachypnea without respiratory failure. The human airway epithelium contains highly specialized pulmonary neuroendocrine cells (PNEC) system. It's function remains unknown but is hypothysed to act in modulation of fetal lung growth and in post-natal stem cell condition [199]. The PNEC system permits synthesis and release of serotonin and neuropeptides such as bombesin [200]. As normal bombesin levels decrease after mid-gestation, its overexpression in NCHI could be attributed to a non-regression of neuroendocrine cells [201]. Clinical presentation is typically a respiratory distress in post-natal young infant (mean age 3.8 months in a large serie, but cases in older children have been reported [202]. HRCT shows patchy centrally ground-glass opacifications and air trapping [203]. On lung biopsy, the histological abnormality is hyperplasia of neuroendocrine cells within bronchioles documented by bombesin immunohistochemistry. The follow-up reveals in some cases the persistence of tachypnea and oxygen requirement for several months. Usually, there is a good prognosis [7,8,196,202].

Chronic pneumonitis in infancy was first described by Katzenstein et al. [4]. The clinical and radiologic features are similar to those observed in other forms of ILD. Specific histologic abnormalities include diffuse thickening alveolar septa, hyperplasia of type 2 AEC, and presence of primitive mesenchymal cells within the alveolar septa. In some cases, foci of pulmonary proteinosis-like material have been observed in air spaces. The prognosis has been reported to be poor with a high mortality rate.

Other disorders associated with pulmonary development and growth abnormalities encompass a broader spectrum of respiratory manifestations and are more adequately integrated in the classification of diffuse lung diseases [8].

## Treatment and outcome

### General measures

Management of children with ILD includes administration of oxygen for chronic hypoxaemia, and maintenance of nutrition with an adequate energy intake, Immunization with influenza vaccine on an annual basis is recommended along with other routine immunizations against major respiratory pathogens [11]. In addition, aggressive treatment of intercurrent infections and strict avoidance of tobacco smoke and other air pollutants are strongly recommended.

### Pharmacologic therapy

A very few children do not require any treatment and recover spontaneously. In the majority of cases, treatment with immunosuppressive, anti-inflammatory, or anti-fibrotic drugs is required for weeks, months or even years [1,9,61]. Various drugs discussed below can be used, but no guidelines for treatment of ILD in children have been proposed so far. The major reason is the very limited number of pediatric patients available for a prospective clinical trial. In addition, controlled studies with a placebo arm are unacceptable because of the poor prognosis of untreated cases and the reported efficacy of anti-inflammatory therapies in a number of pediatric ILD.

At the present time, the main therapeutic strategy is based on the concept that suppressing inflammation may most likely prevent progression to fibrosis. Among the anti-inflammatory agents used in pediatric ILD, steroids are the preferred choice, administered orally and/or intravenously. This has been well illustrated by the results of the ERS Task Force on pediatric ILD [9]. Oral prednisolone is most commonly administered at a dose of 1-2 mg/kg/day [1]. Children with significant disease are best treated with pulsed methylprednisolone at least initially [61,204]. This is usually given at a dose of 10-30 mg/kg/day for 3 days consecutively at monthly intervals. The minimum number of cycles recommended is 3 but treatment may need to be continued for a longer period of 6 months or more depending on response. When the disease is under control, the dosage of methylprednisolone can be reduced or the time between cycles can be spaced out. The disease may then be controlled with oral prednisolone preferably given as an alternate day regime. In few cases oral prednisolone is used from the beginning simultaneously with intravenous methylprednisolone but this is only recommended in those with very severe disease. Methylprednisolone may be effective when other forms of steroids administration fail without significant side effects.

An alternative to steroids is hydroxychloroquine with a recommended dose of 6-10 mg/kg/day. Individual case reports have described a response to hydroxychloroquine even in the presence of steroid resistance [1,205,206]. Some groups have proposed to base the decision as to which agent to use on the lung biopsy findings, with a preference for steroids in case of large amount of desquamation and inflammation and for hydroxychloroquine if increased amounts of collagen representing pre-fibrotic change are found. However, as documented in the ERS Task Force on pediatric ILD, the preferred choice between steroids or hydroxychoroquine in children is highly dependent on the expertise of the center in charge of the patient, and does not seem to be oriented by the histopathological pattern [9]. In case of severe disease, steroids and hydroxychloroquine may be associated. In situations of inefficiency of steroids and hydroxychloroquine, other immunosuppressive or cytotoxic agents such as azathioprine, cyclophosphamide, cyclosporine, or methotrexate may be used. These treatments have been used mainly in situation of autoimmune disorders.

Promising therapeutic options include macrolides. Indeed, these antibiotics have been shown to display a number of anti-inflammatory and immunomodulatory actions. Although the mechanisms and cellular targets specific to macrolide activity remain to be elucidated, beneficial effects in several chronic lung diseases including chronic obstructive pulmonary diseases (COPD) and cystic fibrosis have been reported [207,208]. Of interest is the ability of macrolides to accumulate in host cells including epithelial cells and phagocytes. In a recent report, a favorable response to treatment with clarithromycine has been described in an adult patient with DIP [209]. Other new therapeutic strategies currently proposed in adult patients target fibrogenic cytokines. The Th1 cytokine interferon-γ has an antifibrotic potential through suppression of Th2 fibrogenic functions. Antagonists to TGF-β include pirfenidone and decorin. The use of molecules directed against TNF-α such as the soluble TNF-α receptor agent etanercept is also under investigation. To date, there are no reports on the use of these novel therapies in pediatric ILD. Finally, in the coming years, it is likely that an expanding number of molecules aimed at favoring alveolar surface regeneration and repair through activation and proliferation of tissue-resident (progenitor) cells will come out.

### Other specific treatment strategies

Depending on the underlying diseases, several specific treatment strategies needs to be considered. These include whole lung lavage for pulmonary alveolar proteinosis, which has been reported to be effective by removing the material from the alveolar space [210]. GM-CSF has also been shown of interest in this disease [171]. Other strategies such as interferon-α for pulmonary haemangiomatosis are effective [211].

In recent years, lung transplantation has emerged as a viable option in children of all ages, even in young infants, and lung or heart-lung transplantation may be offered as an ultimate therapy for end-stage ILD [11]. The outcome and survival do not seem to be different from those reported in conditions others than ILD, although comparisons are difficult to establish due to the limited number of reported cases.

### Outcome

Response to treatment and outcome can be evaluated in children based on several criteria such as decrease in cough and dyspnea, increase in oxygenation at rest and sleep, and changes in pulmonary function tests [1,11]. Improvement on thoracic HRCT may also be seen, but tends to occur over a much longer period of time. Reports in pediatric ILD have not shown a good correlation between histological findings and outcome. Some children with relatively severe fibrosis on lung biopsy make good progress, whereas others with mild desquamation have a poor outcome. This is probably due to the variable severity of the disease in different parts of the lung especially in relation to the particular area biopsied, despite HRCT guidance. Overall a favorable response to corticosteroid therapy can be expected in 40-65% of cases, although significant sequelae such as limited exercise tolerance or the need for long-term oxygen therapy are often observed. Reported mortality rates are around 15%. The outcome for infants is more variable [1,61].

## Conclusion

Pediatric ILD comprises a large spectrum of disorders, with compelling evidence that some of these disorders are observed more frequently in infants, while others are more specific to older children. Ongoing basic research will provide new insights into the molecular basis of ILD pathogenesis (including genetic factors causing familial disease) in children, and is expected to identify important preclinical markers of disease, pathways of disease regulation, and novel potential targets for therapeutic intervention. For the future, there is a strong need for international collaboration which will allow collecting sufficiently large cohorts of patients with specific entities in order to perform proper therapeutic trials. As a prerequisite, however, a clear and standardised classification of the histopathology of the underlying conditions is critical. Such multicenter trials will help to reduce the still considerable morbidity and mortality in children with ILD.

## Abbreviations

(ARDS): Acute respiratory distress syndrome; (AEC): Alveolar epithelial cells; (ATS): Amercican Thoracic Society; (AS): Ankylosing spondylitis; (Ab): Antibodie; (anti-CCP): Anticyclic citrullinated peptide; (anti-GBM): Anti-glomerular basement membrane;(Jo1): Anti-histidyl-t-RNA synthetase; (ANCA): Anti-neutrophil cytoplasmic antibody; (ANA): Antinuclear antibodies; (anti-U1-RNP): Anti-U1-ribonucleoprotein; (SaO2): Arterial oxygen saturation; (ABCA3): ATP-binding cassette, sub-family A, member 3; (BiP): Binding immunoglobulin protein; (BAL): Bronchoalveolar lavage; (CASR): Calcium-sensing receptor; (CGD): Chronic granulomatous disease; (COPD): Chronic obstructive pulmonary disease; (CSS): Churg-Strauss syndrome; (CTD): Connective tissue disorders; (CMV): Cytomegalovirus; (c): Cytoplasmic-staining; (DIP): Desquamative interstitial pneumonia; (DAD): Diffuse alveolar damage; (DAH): Diffuse alveolar hemorrhage; (DLCO): Diffusing capacity of the lung for carbon monoxide; (ER): Endoplasmic reticulum; (ET): Endothelin; (EMT): Epithelial-mesenchymal transition; (EBV): Epstein-Barrr virus; (ERS): European Respiratory Society; (FRC): Functional residual capacity; (*SFTPB*): Gene coding for SP-B; (*SFTPC*): Gene coding for SP-C; (GM-CSF): Granulocyte/macrophage colony-stimulating factor; (HSP): Henoch-Schönlein purpura; (HRCT): High-resolution computed tomography; (HIV): Human immunodeficiency virus; (HP): Hypersensitivity pneumonitis; (Ig): Immunoglobulin; (ILD): Interstitial lung disease; (KL-6): Kerbs von Lungren 6; (LIP): Lymphocytic interstitial pneumonia; (MMP): Metalloproteinases; (MPA): Microscopic polyangiitis; (MCTD): Mixed connective tissue disease; (NCHI): Neuroendocrine cell hyperplasia of infancy; (NSIP): Non-specific interstitial pneumonia; (p): Perinuclear-staining; (PAP): Pulmonary alveolar proteinosis; (PFT): Pulmonary function testing; (PIG): Pulmonary interstitial glycogenosis; (PNEC): Pulmonary neuroendocrine cells; (RV): Residual volume; (RSV): Respiratory syncitial virus; (RA): Rheumatoid arthritis; (RNP): Ribonucleoprotein; (SRP): Signal recognition particle; (SS): Sjögren syndrome; (Sm): Smith antigen; (SP): Surfactant proteins; (SLE): Systemic lupus erythematosus; (SSc): Systemic sclerosis; (TTF-1): Thyroid transcription factor 1; (TLC): Total lung capacity; (TLCO): Transfer factor of the lung for carbon monoxide; (TGF): Transforming Growth Factor; (UIP): Usual interstitial pneumonia; (WG): Wegener's granulomatosis;

## Competing interests

The authors declare that they have no competing interests.

## Authors' contributions

AC and NN contributed equally to this work and should be considered as joint first authors. AC, NN and HC drafted the review. RE and BF have been involved in revising critically the review. All authors read and approved the final manuscript.
